# Novel Co_5_ and Ni_4_ Metal Complexes and Ferromagnets by the Combination of 2-Pyridyl Oximes with Polycarboxylic Ligands

**DOI:** 10.3390/molecules27154701

**Published:** 2022-07-22

**Authors:** Foteini Dimakopoulou, Costantinos G. Efthymiou, Ciaran O’Malley, Andreas Kourtellaris, Eleni Moushi, Anastasios Tasiopooulos, Spyros P. Perlepes, Patrick McArdle, Ernesto Costa-Villén, Julia Mayans, Constantina Papatriantafyllopoulou

**Affiliations:** 1 School of Biological and Chemical Sciences, College of Science and Engineering, National University of Ireland Galway, University Road, H91 TK33 Galway, Ireland; f.dimakopoulou1@nuigalway.ie (F.D.); dinosef@yahoo.com (C.G.E.); c.omalley16@nuigalway.ie (C.O.); patrick.mcardle@nuigalway.ie (P.M.); 2 Department of Chemistry, University of Cyprus, Nicosia 1678, Cyprus; kourtellaris.andreas@ucy.ac.cy (A.K.); atasio@ucy.ac.cy (A.T.); 3 Department of Life Sciences, European University of Cyprus, Nicosia 2404, Cyprus; e.moushi@euc.ac.cy; 4 Department of Chemistry, University of Patras, 26504 Patras, Greece; perlepes@upatras.gr; 5 Departament de Química Inorgànica i Orgànica, Secció Inorgànica and Institute of Nanoscience (IN2UB) and Nanotecnology, Universitat de Barcelona, Marti i Franques 1-11, 08028 Barcelona, Spain; ernesto.costa@qi.ub.edu (E.C.-V.); julia.mayans@qi.ub.edu (J.M.)

**Keywords:** coordination polymers, metal complexes, carboxylates, magnetic properties, pyridyl oximes, nickel, cobalt

## Abstract

The use of 2-pyridyl oximes in metal complexes chemistry has been extensively investigated in the last few decades as a fruitful source of species with interesting magnetic properties. In this work, the initial combination of pyridine-2-amidoxime (pyaoxH_2_) and 2-methyl pyridyl ketoxime (mpkoH) with isonicotinic acid (HINA) and 3,5-pyrazole dicarboxylic acid (H_3_pdc) has provided access to three new compounds, [Ni_4_(INA)_2_(pyaox)_2_(pyaoxH)_2_(DMF)_2_] (**1**), [Co_5_(mpko)_6_(mpkoH)_2_(OMe)_2_(H_2_O)](ClO_4_)_6_ (**2**), and [Co_5_(OH)(Hpdc)_5_(H_2_pdc)] (**3**). **1** displays a square-planar metal topology, being the first example that bears simultaneously HINA and pyaoxH_2_ in their neutral or ionic form. The neighbouring Ni_4_ units in **1** are held together through strong intermolecular hydrogen bonding interactions, forming a three-dimensional supramolecular framework. **2** and **3** are mixed-valent Co_4_^III^Co^II^ and Co_2_^III^Co^II^_3_ compounds with a bowtie and trigonal bipyramidal metal topology, accordingly. Direct current and alternate current magnetic susceptibility studies revealed that the exchange interactions between the Ni^II^ ions in **1** are ferromagnetic (*J* = 1.79(4) cm^−1^), while **2** exhibits weak AC signals in the presence of a magnetic field. The syntheses, crystal structures, and magnetic properties of **1–3** are discussed in detail.

## 1. Introduction

Polynuclear metal complexes are hybrid metal–organic compounds, in which metal ions are held together through inorganic or organic ligands forming discrete species of zero dimensionality [1,2,3,4]. The synthesis and characterisation of such species have attracted an immense research interest the recent decades due to their potential applications in the environmental, biomedical, technological and industrial field [5,6,7,8,9,10,11,12,13,14]. In particular, polynuclear metal compounds often display unique structural features and combine physical properties (magnetism, luminescence, etc.) that cross the boundaries between a variety of research areas, including bioinorganic chemistry, drug discovery, catalysis, molecular magnetism, and others. In regard to the latter, the discovery that the paramagnetic metal complexes have the potential to possess magnetic properties in the absence of an external magnetic field at very low temperature, i.e., exhibiting single molecule magnetism (SMM) behaviour, marked the commencement of a new era in the field of molecular nanomaterials [5,6,7]. The magnetic behaviour of SMMs results from the combination of a large ground spin state (*S*) with a large and negative easy-axis type of magnetoanisotropy as measured by the axial zero-field splitting parameter, *D.* Such species often display extraordinary properties at the nanoscale level, e.g., quantum tunnelling of magnetisation and quantum phase interference, and they are excellent candidates in data storage devices applications, quantum computers, imaging, etc. [15,16,17,18,19,20]. On the other hand, metal complexes with small magnetic anisotropy have the potential to be used as magnetic refrigerants in magnetic refrigeration, which is an environmentally friendly technique for refrigeration at ultra-low temperatures [8,9,10].

The connection of metal complexes through polytopic organic linkers leads to the formation of coordination polymers. In the case of 1D coordination polymers consisting of paramagnetic building units, strong exchange interactions might be present along the chain, which gives rise to another family of magnetic materials, namely single-chain magnets (SCMs) [21,22,23]. SCMs are excellent candidates for applications similar to those of SMMs; furthermore, they have been investigated as molecular ferromagnets, synthetic metallic conductors, non-linear optical or ferroelectric materials [24,25,26]. As the dimensionality of the network increases, the accessible porosity of the material increases too, leading to porous coordination polymers known as metal–organic frameworks (MOFs) [27,28,29]. MOFs are of special interest as they display appealing structural features, such as large surface area and tuneable porosity, which make them suitable for the adsorption of large guest molecules. The porosity is often combined with magnetism and/or another physical property, e.g., photoluminescence, etc. The synergistic effect between different properties may enhance the performance of such species in applications related to sensing, catalysis, drug delivery, spintronics, photonics, etc. [30,31,32,33,34,35,36,37].

**Scheme 1:**
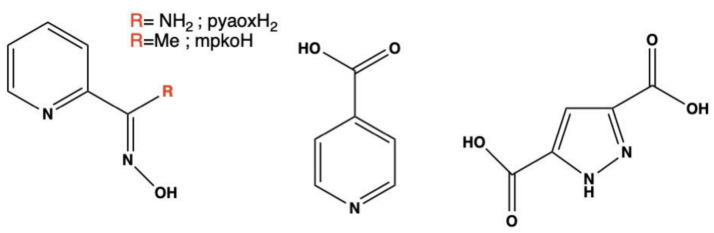
Schematic representation of 2-pyridyl oximes (**left**), isonicotinic acid (**centre**) and 3,5-pyrazole dicarboxylic acid (**right**).

The structural and physical properties of the discrete metal complexes and coordination polymers are strongly affected by the nature of the metal ion(s) and the ligand(s) present in their structures. The organic linkers possess suitable sites for metal coordination, and they provide the desirable stability through the formation of hydrogen bonding, π–π stacking, and other intermolecular interactions. Such interactions are significant, and they are even more profound when metal complexes are linked to form coordination polymers or MOFs, as they can introduce structural flexibility and affect the MOF selectivity toward specific guest molecules [38]. One family of ligands that has been intensively explored for the synthesis of metal compounds with interesting magnetic properties is the 2-pyridyl oximes; 2-pyridyl oximes have the general formula (py)C(R)NOH (Scheme 1), with the electronic properties and functionality of the R group playing a crucial role on the coordination properties of the ligands. (py)C(R)NOH have been key “players” in several areas of single-molecule and single-chain magnetism, as they tend to link a large number of metal ions, favouring also the presence of ferromagnetic exchange interactions between the metal centres [39,40,41,42,43,44,45,46,47,48,49]. Although the employment of 2-pyridyl oximes has led to significant breakthroughs in the areas of molecular magnetism, their use in the field of MOFs remains limited. To this end, our group has been investigating the ligand blend 2-pyridyl oximes (e.g., pyridine-2-amidoxime and 2-methyl pyridyl ketoxime) and polycarboxylates, which has led to the isolation of the first 2-pyridyl oxime-based MOFs, and several coordination polymers [50,51,52]. Some of the MOFs display unprecedented metal topologies and metal ion encapsulation capability. A representative such example is [Cu_4_(OH)_2_(pma)(mpko)_2_]_n_, pma^4−^ = the tetra-anion of 1,2,4,5-benzene tetracarboxylic acid, and mpko^−^ = the anionic form of 2-methyl pyridyl ketoxime, which exhibits a good Fe^III^ adsorption capacity; interestingly, the magnetic properties of the latter are strongly related to the amount of the encapsulated Fe^III^ ions [52].

With the above in mind, we decided to explore further the reaction mixture of 2-pyridyl oximes and polycarboxylic acids, and, in particular, we introduced isonicotinic acid (pyridine-4-carboxylic acid) [53,54,55,56,57,58,59,60,61,62,63,64,65] and 3,5-pyrazole dicarboxylic acid [66,67,68,69,70,71,72,73,74,75,76,77,78,79,80,81,82,83,84,85] to the reaction system (Scheme 1). Both carboxylic acids have been extensively investigated in the field of coordination compounds, and they have been proven a fruitful source of discrete metal complexes and coordination polymers; [53,54,55,56,57,58,59,60,61,62,63,64,65,66,67,68,69,70,71,72,73,74,75,76,77,78,79,80,81,82,83,84,85] yet their combination with oximic ligands remains unexplored. Herein, we report three new Ni_4_ (**1**), Co^III^_4_Co^II^ (**2**), and Co^III^_2_Co^II^_3_ (**3**) complexes from this ligand combination. The syntheses, crystal structures and magnetic properties of these compounds have been studied, revealing that **1** is a ferromagnet being the first example bearing both pyaoxH_2_ and HINA. **3** displays an unprecedented {Co^II^_3_(μ_3_-OH)}^7+^ planar unit which can be used as a model for the study of spin frustration in triangular Co^II^_3_ systems.

## 2. Results and Discussion

### 2.1. Synthetic Discussion

Our group has been exploring the employment of 2-pyridyl oximes in combination with polycarboxylic acids toward the synthesis of new metal compounds. The latter involved experiments with the use of benzene-1,4-dicarboxylic acid, benzene-1,3,5-tricarboxylic acid and benzene-1,2,4,5-tetracarboxylic acid [50,51,52]. The initial promising results prompted us to explore further this reaction system by using different carboxylic ligands, such as isonicotinic acid (HINA) and 3,5-pyrazole dicarboxylic acid (H_3_pdc). To this end, a wide range of experiments was conducted, aiming at studying the effect of different synthetic parameters (temperature, presence/absence or kind of base, metal ratio of the reactants, metal sources, etc.) on the identity and crystallinity of the isolated product(s) and the yield of the reaction.

The reaction mixture of Ni(ClO_4_)_2_·6H_2_O/pyaoxH_2_/HINA (2:4:1.5) in DMF at 110 °C gave a dark brown solution from which dark brown crystals of [Ni_4_(INA)_2_(pyaox)_2_(pyaoxH)_2_(DMF)_2_] (**1**) were subsequently isolated in good yield. The stoichiometric equation of the reaction that led to the formation of **1** is represented in Equation (1).

(1) $$4\mathrm{Ni}({\mathrm{ClO}}_{4}{)}_{2}\cdot 6H_{2}O+4{\mathrm{pyaoxH}}_{2}+2\mathrm{HINA}+2\mathrm{DMF}+8{\mathrm{Et}}_{3}N\overset{DMF}{\underset{}{\to }}\;[{\mathrm{Ni}}_{4}(\mathrm{INA}{)}_{2}(\mathrm{pyaox}{)}_{2}(\mathrm{pyaoxH}{)}_{2}(\mathrm{DMF}{)}_{2}]+8{{\mathrm{ClO}}_{4}}^{-}+8{{\mathrm{HNEt}}_{3}}^{+}+24H_{2}O\;1$$

The kind of base and/or metal source does not have any impact on the identity of the isolated compound, but it affects its crystallinity. The next step included the replacement of Ni^II^ by Co^II/III^ wishing to shed light on how the type of the metal ion affects the identity of the product. Similar reactions that led to **1** were performed by using a Co^II^ source instead of Ni, which, in their vast majority, led to the isolation of previously reported single-ligand Co^II^ MOFs based on INA^−^ [53,54]. Likewise, the use of mpkoH instead of pyaoxH_2_ provided access to compounds that contain INA^−^. This prompted us to perform reactions with an excess of the oximic ligand, which led to the new mixed-valent cationic pentanuclear compound [Co^III^_4_Co^II^(mpko)_6_(mpkoH)_2_(OMe)_2_(H_2_O)](ClO_4_)_6_ (**2**), while the use of H_3_pdc in the place of HINA led to the isolation of the new neutral pentanuclear compound [Co_5_(OH)(Hpdc)_5_(H_2_pdc)] (**3**). The stoichiometric equations of the reaction that describe the formation of **2** and **3** are shown in Equations (2) and (3).

(2) $$5\mathrm{Co}({\mathrm{ClO}}_{4}{)}_{2}\cdot 6H_{2}O+8\mathrm{mpkoH}+O_{2}+4{\mathrm{NaO}}_{2}\mathrm{CMe}+2\mathrm{MeOH}\overset{}{\underset{}{\to }}\;[{\mathrm{Co}}_{5}(\mathrm{mpko}{)}_{6}(\mathrm{mpkoH}{)}_{2}(\mathrm{OMe}{)}_{2}(H_{2}O)]({\mathrm{ClO}}_{4}{)}_{6}+4{\mathrm{Na}}^{+}+4{{\mathrm{ClO}}_{4}}^{-}+4{\mathrm{MeCO}}_{2}H+31H_{2}O\;2$$

(3) $$5\mathrm{Co}(O_{2}\mathrm{CMe}{)}_{2}\cdot 4H_{2}O+6H_{3}\mathrm{bdc}+1/2O_{2}+10{\mathrm{Et}}_{3}N\overset{DMF}{\underset{}{\to }}\;[{\mathrm{Co}}_{5}(\mathrm{OH})(\mathrm{Hpdc}{)}_{5}(H_{2}\mathrm{pdc})]+10{{\mathrm{HNEt}}_{3}}^{+}+10{{\mathrm{MeCO}}_{2}}^{-}+20H_{2}O\;3$$

### 2.2. Description of Structures

**Figure 1:**
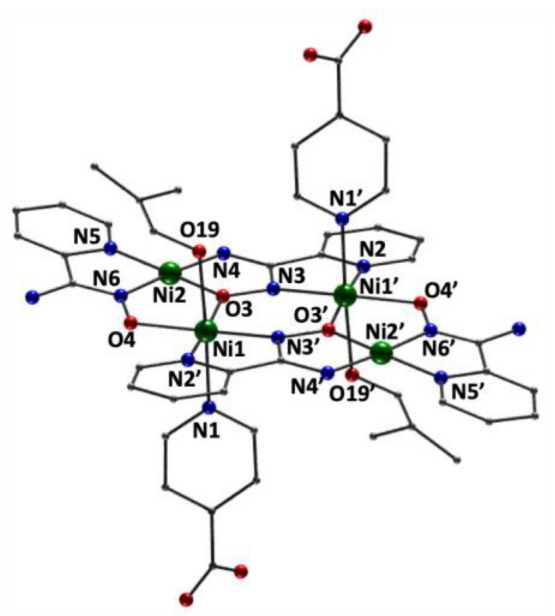
Representation of the molecular structure of **1**. Colour code: Ni, green; N, blue; O, red; C, grey.

**Figure 2:**
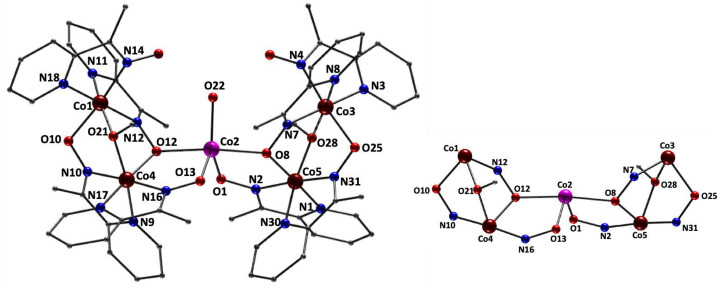
Representation of the molecular structure of **2** (**left**) and the bowtie metal topology of its structural core (**right**). Colour code: Co^II^, pink; Co^III^, dark red; N, blue; O, red; C, grey.

**Figure 3:**
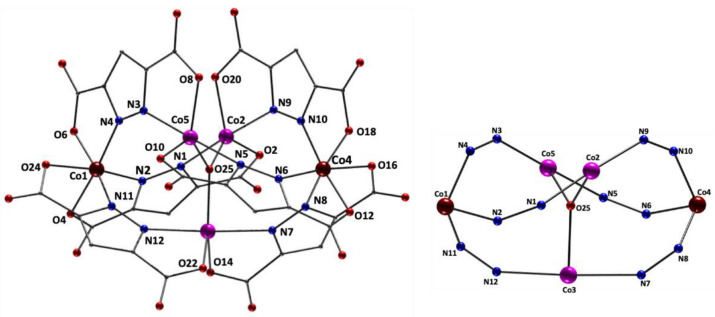
Representation of the molecular structure of **3** (**left**) and the trigonal pipyramidal metal topology of its structural core (**right**). Colour code: Co^II^, pink.; Co^III^, dark red; N, blue; O, red; C, grey.

Representations of the molecular structures of **1–3** are shown in Figure 1, Figure 2 and Figure 3. Selected interatomic distances and angles are listed in Tables S1–S3.

**Scheme 2:**
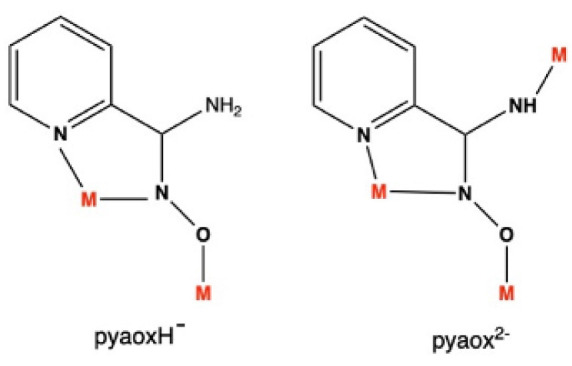
The coordination modes of the pyaox^2−^ and pyaoxH^−^ ligands in **1**.

**1** crystallizes in the monoclinic space group P2_1_/c. Its structure is based on neutral, centrosymmetric [Ni_4_(INA)_2_(pyaox)_2_(pyaoxH)_2_(DMF)_2_] units (Figure 1) that are held together through strong hydrogen bonding interactions forming a 3D network (Figure S1). The Ni_4_ compound in **1** is composed of two distorted octahedral (Ni1 and its symmetry equivalent) and two square planar Ni^II^ atoms (Ni2 and its symmetry equivalent), which are connected through two η^1^:η^1^:η^1^:μ pyaoxH^-^, and two η^1^:η^1^:η^1^:η^2^:μ_3_ pyaox^2-^ ligands (Scheme 2). The coordination sphere in Ni1 and Ni1’ is completed by one terminally ligated DMF molecule and one monodentate INA^−^ ion. The four metal centres in **1** are co-planar.

The carboxylate group of the deprotonated INA^−^ ligand forms strong intermolecular hydrogen bonding interactions with the neutral and/or deprotonated amino groups from neighbouring Ni_4_ species: (N7···O1 = 2.848 Å, H7B···O1 = 2.029 Å, N7-H7B···O1 = 158.92°; N7···O2 = 3.005 Å, H7A···O2 = 2.197 Å, N7-H7A···O2 = 156.46°; N4···O1 = 3.053 Å, H1N4···O1 = 2.186 Å, N4-H1N4···O1 = 171.52°). These intermolecular interactions along with the fact that that neighbouring Ni_4_ units are in close proximity (Ni1 … Ni2 = 7.7 Å) result in the formation of a three-dimensional supramolecular network. The N7-H7A···O2 and N4-H1···O1 H-bonds favour the formation of a 2D supramolecular network (Figure S2), whereas the Ni_4_ complexes lie roughly on the same plane and each complex is surrounded by six neighbouring Ni_4_ units in a hexagonal topological arrangement. Neighbouring 2D planes are arranged in a parallel fashion, being interconnected through the N7-H7B···O1 H-bond, leading to the formation of a 3D supramolecular network (Figure 1, right), which can be described as a pseudo 3D polymer or as a 3D supramolecular framework. It is worth mentioning that **1** displays significant thermal stability (Figure S3), which is a result of its pseudo-polymeric nature. In particular, there is a mass loss of ca 2% below 100 °C, which can be attributed to adsorbed humidity; this is then followed by a plateau with the step at 350 °C corresponding to the compound breakdown.

The tetranuclear {Ni_4_} single-decker core of compound **1** has been found previously as a fundamental building unit in a couple of multiple-decker based polynuclear Ni complexes. Being composed by three and four {Ni_4_} layers/deckers, respectively, Ni_12_ and Ni_16_ complexes can be assumed as the trimer and the tetramer version of **1** [40,41]. Both Ni_12_ and Ni_16_ are ferromagnets bearing a high-spin ground state of S = 6 and S = 8, respectively. Ferromagnets are a special category of magnetic materials with their properties being derived by the ferromagnetic exchange coupling among the paramagnetic centres. They are candidates for interesting potential applications, such as spintronics, magnetic coolers, etc.

**2** crystallizes in the triclinic space group *P*_−1_. Its structure consists of [Co^II^Co^III^_4_(mpko)_6_(mpkoH)_2_(OMe)_2_(H_2_O)] metal complexes and ClO_4_^-^ counterions (Figure 2). The five metal ions are held together through four η^1^:η^1^:η^1^:μ mpko^-^ and two η^2^:η^1^:η^1^:μ_3_ mpko^-^ ions, forming a distorted bowtie topology. Furthermore, two μ-MeO- ions bridge the Co^III^ ions, i.e., Co3 and Co5, and Co1 and Co4, respectively. Alternatively, the structure of **2** can be described as consisting of a central {Co^II^(mpko)_4_(H_2_O)}^2+^ unit on both sides of which are located two dinuclear {Co^III^_2_(mpko)_3_(mpkoH)}^3+^ moieties.

Co1 and Co3–Co5 are six-coordinate with an octahedral coordination geometry. Co2 is five-coordinate displaying trigonal bipyramidal geometry (*τ* = 0.77) [86]. The coordination spheres of Co1 and Co3 are completed by two neutral chelate mpkoH ligands, while one terminally ligated H_2_O molecule completes the coordination sphere of Co2. The Co_5_ cations in **2** are in proximity with the shortest metal∙∙∙metal distance between the neighbouring Co_5_ units being 6.537 Å (Co3∙∙∙Co5). The crystal structure of **2** is stabilised through strong intramolecular hydrogen bonding interactions between the terminally ligated water molecule (O22, donor) and the oximic groups (O6, O15), which act as acceptors: O22···O6 = 2.589 Å, H6···O22 = 2.041 Å, O6-H6···O22 = 123.75°; O22···O2 = 2.644 Å, H2···O22 = 2.052 Å, O2-H2···O22 = 128.87

**3** crystallizes in the monoclinic space group I2/a consisting of mixed-valent pentanuclear compounds [Co^III^_2_Co^II^_3_(OH)(Hpdc)_5_(H_2_pdc)] (Figure 3, left). The five metal centres in **3** are held together through a μ_3_-OH- ion and six η^1^:η^1^:η^1^:η^1^:μ carboxylate ligands. The former link Co2, Co3 and Co5, forming a plane above and below of which the remaining two metal ions are located; hence, the structural core of **3** possesses a trigonal bipyramidal metal topology with the distance of Co1 and Co4 from the equatorial plane being 3.848 Å and 3.896 Å, correspondingly (Figure 3, right).

Co1 and Co4 are six-coordinate with an octahedral coordination geometry. Co2, Co3 and Co5 are five coordinate displaying trigonal bipyramidal geometry (*τ* = Co2, 0.83; Co3, 0.76; Co5, 0.77) [86]. The Co_5_ molecules in **3** are relatively in close proximity with the shortest metal∙∙∙metal separation between the adjacent Co_5_ units being 7.937 Å (Co4∙∙∙Co4). **3** is the first example of a Co compound bearing H_3_pdc in its neutral or anionic form, being also one of the highest nuclearity discrete metal complexes with this ligand.

### 2.3. Magnetism Studies

**Figure 4:**
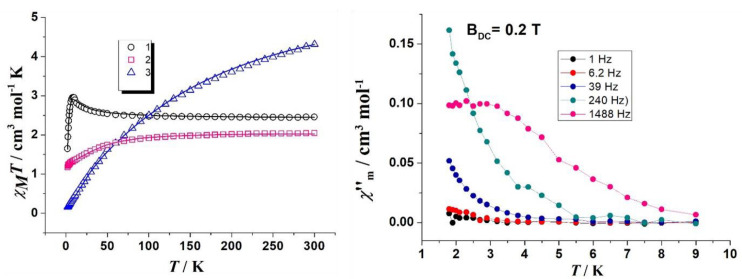
(**Left**) *χ_M_T* vs. T plots for complexes **1** (black circles), **2** (pink squares) and **3** (blue triangles). Solid line represents the best fit. (**Right**) *χ_M_*″ vs. *T* plot for complex **2** at different frequencies under a dc field of 0.2 T.

Direct current magnetic susceptibility measurements (DC) were carried out on samples of **1**, **2** and **3** in the 2–300 K temperature range and under a field of 0.03 T (1.28 MHz), and they are plotted as *χ*_M_*T* vs. *T* plot. (Figure 4). The *χ*_M_*T* values at room temperature (2.30, 1.95 and 4.31 cm^3^ mol^−1^ K for **1**, **2** and **3**, respectively) are very close to the theoretical spin-only value of 2.00, 1.875 and 5.625 cm^3^ mol^−1^ K corresponding to two Ni^II^, one Co^II^ and three Co^II^ non-interacting cations, respectively.

Complex **1** is a tetranuclar Ni^II^ system, in which Ni2 and Ni2’ present a square-planar geometry being diamagnetic, hence, magnetically, the molecule behaves as a dinuclear Ni^II^ complex. The *χ_M_T* vs. *T* curve for **1** shows a weak ferromagnetic interaction between the Ni^II^ paramagnetic cations, reaching a value of 2.96 cm^3^ mol^−1^ K at 9 K; then, there is an abrupt decay until it reaches to a minimum value of 1.64 cm^3^ mol^−1^K at 2 K, due to weak intermolecular interactions or anisotropy effects. The curve has been fitted using the Hamiltonian *Ĥ* = -2*J* (S_Ni1_·S_Ni1’_) + *DS_z_* + Σμ*g_eff_HS* yielding in the best fitting values of *J* = 1.79(4) cm^−1^, *D_ion_* = 2.48(2) cm^−1^ and *g* = 2.20 using PHI software [87]. This is in perfect agreement with previously reported multiple decker Ni complexes based on Ni_4_ layers that exhibit similar magnetic exchange pathways [40,41].

Complex **2** is a pentanuclear Co compound with only one paramagnetic Co^II^ being present in the molecule; thus, it behaves as a mononuclear compound. The *χ_M_T* decay when lowering temperature is due to the axial zero field splitting of the Co^II^ cation evaluated as *D_ion_* = 47.7(7) cm^−1^ and *g* = 2.085(2). The relatively high value of *D* parameter agrees with the trigonal bipyramid distorted environment. Alternate-current (AC) measurements were performed in the 1–1488 Hz frequency range, and weak tails were observed (Figure 4, right) in the presence of a magnetic field of 0.2 T, which is indicative of the single-ion magnetism behaviour for **2**. However, this AC response is too weak to extract any relaxation parameter.

Complex **3** possesses an uncommon magnetic core: usually, *μ_3_*-OR cobalt trimers present a defective-cubane topology, whereas **3** is a *μ_3_*-O tricobalt complex where the oxo donor and the three cobalt atoms are co-planar, defining a triangular arrangement of cations with the three Co-O-Co bond angles being very similar (~120°). A CCDC search does not reveal any similar structure for which the magnetic study has been performed, and it makes complex **3** a quite interesting study case for the spin-frustrated Co^II^ triangle. The *χ_M_T* curve for **3** reveals an antiferromagnetic coupling between the three Co^II^ cations; it continuously decreases with decreasing temperature until it reaches the value of 0.200 cm^3^ mol^−1^ K at 2 K. The used Hamiltonian was *Ĥ* = −2*J* (*S*_Co2_·*S*_Co3_+ *S*_Co3_·*S*_Co5_+ *S*_Co2_·*S*_Co5_) + *DS_z_*+Σμ*g_eff_HS*, yielding in the fitting parameters *J* = −14.18(1) cm^−1^, *g* = 2.075(3) and *D* = 10.55(4) cm^−1^.

## 3. Materials and Methods

### 3.1. Materials, Physical, and Spectroscopic Measurements

All the manipulations were performed under aerobic conditions using materials (reagent grade) and solvents as received. Nickel(II) perchlorate hexahydrate (CAS: 13520-61-1), Isonicotinic acid 99% (CAS: 55-22-1), 3,5 pyrazole dicarboxylic acid monohydrate 97% (CAS: 303180-11-2), Cobalt(II) acetate tetrahydrate 98% A.C.S.(CAS: 6147-53-1) and cobalt(II) chloride hexahydrate 98% (CAS: 7791-13-1) were obtained as received from Sigma-Aldrich. Dimethylformamide > 99.5% HPLC grade (68-12-2) was purchased from Fluka and triethylamine (CAS: 121-44-8) was obtained from Sigma-Aldrich. mpkoH and pyaoxH_2_ was prepared as described elsewhere [88,89]. WARNING: *Perchlorate salts are potentially explosive; such compounds should be used in small quantities and treated with utmost care at all times*.

Elemental analysis (C, H, N) was performed by the in-house facilities of the National University of Ireland Galway, School of Chemistry. Solvothermal synthesis was performed in a Binder oven. IR spectra (4000–400 cm^−1^) were recorded on a Perkin-Elmer Spectrum 400 FT-IR spectrometer. Powder X-ray diffraction was performed with an INEL X-ray diffractometer EQUINOX 0000 using Cu K_a_ radiation (λ = 1.54178 Å, 35 kV, 25 mA). Thermogravimetric analysis (TGA) and differential scanning calorimetry (DSC) were carried out in open aluminium crucibles using an STA625 thermal analyzer (Rheometric Scientific, Piscataway, NJ, USA) with nitrogen being purged in ambient mode. Solid-state, variable-temperature and variable-field magnetic data were collected on single crystals of each sample using an MPMS5 Quantum Design magnetometer operating at 0.03 T in the 300–2.0 K range for the magnetic susceptibility and at 2.0 K in the 0–5 T range for the magnetization measurements. Diamagnetic corrections were applied to the observed susceptibilities using Pascal’s constants.

### 3.2. Compound Synthesis

#### 3.2.1. Synthesis of [Ni_4_(INA)_2_(pyaox)_2_(pyaoxH)_2_(DMF)_2_] (**1**)

Ni(ClO_4_)_2_·6H_2_O (0.073 g, 0.20 mmol) and Et_3_N (114 µL, 0.80 mmol) were added in 5 mL of pyaoxH_2_ (0.054 g, 0.40 mmol) in DMF in a glass vial with a plastic lid. The resultant solution was put in an oven and heated at 110 °C for 1 h during which time the colour of the solution turned dark brown. Then, 5 mL of HINA (0.018 g, 0.15 mmol) were added, and the vial was placed in the oven for a further 48 h. After 2 days, X-ray quality dark red, block-shaped crystals of **1** were observed. The crystals were collected by filtration, washed with DMF and acetone and dried in air. Yield 43%. Anal. Calc. for **1**: C, 43.13; H, 3.96; N, 19.16 Found: C, 43.87; H, 4.01; N, 19.41%. IR data, Figure S3: *ν* (cm^−1^) = 3299 d, 3155 d, 1648 sh, 1600 sh, 1584, 1548 s, 1479 s, 1455 w, 1412 s, 1381 w, 1355 s, 1313 w, 1304 w, 1276 w, 1262 w, 1231 w, 1213 w, 1181 m, 1155 m, 1132 w, 1098 m, 1058 m, 1027 m, 1018 m, 953 m, 864 m, 833 w, 801 m, 781 s, 762 w, 750 w, 731 w, 707 m, 681 s, 669 m, 657 w.

#### 3.2.2. Synthesis of [Co_5_(mpko)_6_(mpkoH)_2_(OMe)_2_(H_2_O)](ClO_4_)_6_ (**2**)

Sodium acetate trihydrate (0.027 g, 0.2 mmol) and 2-pyridyl ketoxime (0.027 g, 0.2 mmol) were dissolved in 15 mL MeOH in a glass vial with a plastic lid. The solution was stirred on a hot plate for 10 min. Co(ClO_4_)_2_·xH_2_O (0.073 g, 0.2 mmol) was then added and the solution turned dark red; it was left under stirring for 30 min further after which it was filtered. The solution was left in an open vial for slow evaporation, and after 2 days, dark red X-ray quality single crystals were observed. Yield 45%. Anal. Calc. for **2***^.^*: C, 36.86; H, 3.98; N, 7.68% Found: C, 36.52; H, 4.23; N, 7.16%. IR data, Figure S4: *ν* (cm^−1^) = 3532 w, 3420 w, 3146 w, 3060 w, 3000 w, 1750 w, 1752 w, 1632 w, 1602 s, 1575 w, 1528 s, 1475 s, 1440 s, 1379 w, 1302 w, 1263 w, 1185 s, 1074 s, 1028 w, 992 w, 931 w, 883 w, 822 w, 774 s, 749 m, 695 s, 655 m.

#### 3.2.3. Synthesis of [Co_5_(OH)(Hpdc)_5_(H_2_pdc)] (**3**)

*Method A*: H_3_pdc (0.034 g, 0.20 mmol), pyaoxH_2_ (0.054 g, 0.40 mmol) and Et_3_N (10 μL mL, 0.07 mmol) were dissolved in 5 mL of DMF in a glass vial with a plastic lid. Co(CH_3_COO)_2_·4H_2_O (0.024 g, 0.10 mmol) was added, and the vial was placed in the oven at 110 °C for 24 h, after which the solution turned brown–pink and X-ray quality purple needles of 2 were formed. The crystals were collected by filtration, washed with DMF and acetone and dried in air. Yield 90%. Anal. Calc. for **3**: C, 29.13; H, 1.14; N, 13.59%. Found: C, 28.74 H, 1.19; N, 13.29%. IR data: *ν* (cm^−1^) = 3544 w, 3421 w, 3124 w, 3040 w, 2791 w, 2476 w, 1622 s, 1602 s, 1494 m,1468 m,1438 w,1412 w, 1371 s, 1306 s, 1259 s, 1097 w, 1059 m,1011 s, 888 w, 841 s, 815 m, 802 w, 777 s, 662 w.

*Method B*: Method A was followed with the only difference being the omission of pyaoxH_2_ from the reaction mixture. The sealed vial was placed in the oven at 110 °C, and after one day, crystalline purple needles of 2 were observed. Yield: 80%

*Method C*: Method A was repeated but using CoCl_2_·6H_2_O (0.023 g, 0.10 mmol) instead of Co(CH_3_COO)_2_·4H_2_O. The vial was left in the oven at 110 °C, and after one day, crystalline purple needles of 2 were observed. Yield: 90%. The product in Methods B and C was identified as **2** by IR spectral comparison with material obtained in method A (Figure S5) and unit cell determination.

### 3.3. Single-Crystal X-ray Crystallography

Single-crystal diffraction data for **1** were collected in an Oxford Diffraction Xcalibur CCD diffractometer using graphite-monochromatic Mo-Kα radiation (λ = 0.71073 Å) at room temperature. Single-crystal diffraction data for **2** and **3** were collected in an Oxford-Diffraction SuperNova diffractometer equipped with a CCD area detector and a graphite monochromator utilising Mo-Kα (for **2**) and Cu-Kα radiation (for **3**). The structures were solved using SHELXT and [90] embedded in the OSCAIL software [91]. The non-H atoms were treated anisotropically, whereas the hydrogen atoms were placed in calculated, ideal positions and refined as riding on their respective carbon atoms. Molecular graphics were produced with DIAMOND [92]. We note that crystal twining occurs in **2**, which affects the quality of the structure. We carried out many experiments in order to grow single crystals of better quality; however, this has not been achieved. We collected three sets of data using two different diffractometers and used the best set to solve the structure.

Unit cell data and structure refinement details are listed in Table 1. The crystal structures have been deposited with the Cambridge Crystallographic Data Centre (CCDC 2180525-2180527), and they can be accessed, free of charge, by filling out the application form at https://www.ccdc.cam.ac.uk/structures/.

## 4. Conclusions

The combination of 2-pyridyl oximes (pyridine-2 amidoxime, H_2_pyaox; 2-methyl pyridyl ketoxime, Hmpko) with isonicotinic acid (HINA) and 3,5-pyrazole dicarboxylic acid (H_3_pdc) provided access to three new Ni^II^ and Co^II/III^ metal complexes. Among them, [Ni_4_(INA)_2_(pyaox)_2_(pyaoxH)_2_(DMF)_2_ (**1**) is a planar centrosymmetric tetranuclear compound that can be characterised as a pseudo-polymer due to the strong hydrogen bonding interactions between the neighbouring discrete units. It is the first reported metal compound that bears both pyaoxH_2_ and HINA in their neutral or anionic form. **2** is a pentanuclear Co^III^_4_Co^II^ complex with a bowtie topology, while **3** is a Co^III^_2_Co^II^_3_ complex with a trigonal bipyramidal metal topology. The three Co^II^ cations in **3** are co-planar with the bridging μ_3_-OH- ion providing a unique spin frustration model for triangular Co^II^_3_ systems.

The magnetic properties of **1–3** have been studied and revealed that there are ferromagnetic interactions between the two paramagnetic Ni^II^ ions in **1** (*J* = 1.79(4) cm^−1^), which is in accordance with previously reported multiple decker Ni_12_ and Ni_16_ compounds based on Ni_4_ repeating units [40,41]. **2** displays weak AC signals in the presence of a magnetic field, while in the case of **3,** the dominant exchange interactions between the metal ions are antiferromagnetic. **3** is a unique example of a OH-centred triangular Co^II^_3_ system in which all the Co atoms and the OH^-^ are co-planar; hence, it is an interesting case study for spin frustration.

Although the isolation of a compound that contains both a 2-pyridyl oxime and isonicotinic acid (HINA) has been successfully achieved in the case of **1**, this has not been the case for 3,5-pyrazole dicarboxylic acid (H_3_pdc). Further studies that include the optimisation of the reaction conditions that will favour the presence of both ligands in the same metal complex, e.g., full deprotonation of ligands, nature of metal ion, etc., are currently in progress and will be reported in the near future.

## Figures and Tables

**Table 1:** Crystallographic data for complexes **1–3**.

Complex	1	2	3
Empirical formula	C_44_H_44_N_14_Ni_4_O_10_	C_116_H_128_Cl_9_Co_10_N_32_O_60_	C_30_H_6_Co_5_N_12_O_25_
Formula weight	1163.77	3838.85	1229.12
Crystal system	Monoclinic	Triclinic	Monoclinic
Space group	*P2_1_/c*	*P*-1	*I2/a*
*a* (Å)	11.6069 (5)	11.28230 (10)	19.1464 (8)
*b* (Å)	12.4541 (5)	15.2083 (2)	29.8272 (10)
*c* (Å)	18.1577 (9)	23.9909 (4)	23.4279 (7)
*α* (^o^)	90	84.1200 (10)	90
*β* (^o^)	107.901 (5)	86.0130 (10)	92.567 (3)
*γ* (^o^)	90	88.3410 (10)	90
*V* (Å^3^)	2497.7 (2)	4083.91 (9)	13,365.9 (8)
*Ζ*	2	1	8
*ρ*_calc_ (g cm^−3^)	1.547	1.561	1.222
μ (mm^−1^)	1.554	1.229	1.014
Measd/independent reflns (*R*_int_)	4549/3473 (0.0492)	14,336/11,846 (0.0301)	11,879/6538 (0.0431)
Parameters refined	326	1020	649
GoF (on *F*^2^)	0.981	1.041	0.956
*R*_1_^a^ (*I* > 2*σ*(*Ι*))	0.0453	0.0939	0.0818
*wR*_2_^b^ (*I* > 2*σ*(*Ι*))	0.1181	0.2691	0.2478
(Δ*ρ*)_max_/(Δ*ρ*)_min_ (e Å^−3^)	0.644/−0.788	3.867/−2.563	0.715/−0.374

^a^*R*_1_ = Σ(|*F*_o_|-|*F*_c_|)/Σ(|*F*_o_|). ^b^ *wR*_2_ = {Σ[*w*(*F*_o_^2^-*F*_c_^2^)^2^]/Σ[*w*(*F*_o_^2^)^2^]}^1/2^.

## Data Availability

Not available.

## Supplementary Materials

The following are available online at https://www.mdpi.com/article/10.3390/molecules27154701/s1, Table S1. Selected interatomic distances (Å) and angles for **1**; Table S2. Selected interatomic distances (Å) and angles for **2**; Table S3. Selected interatomic distances (Å) and angles for **3**; Figure S1. The 2D supramolecular network in **1** coming from the arrangement of Ni4 clusters through H-bonding interactions (left), and the arrangement of two parallel 2D supramolecular planes (right); Figure S2. TGA-DTG (top) and DSC curves (bottom) of compound **1**; Figure S3. IR spectrum of compound **1**; Figure S4. IR spectrum of compound **2**; Figure S5. IR spectra of compound **3**: crystals from method A, top; crystals from method B, middle; crystals from Method C, bottom; Figure S6. Powder XRD patterns of compound **1**: experimental powder pattern, top; simulated, bottom.

## Sample Availability

Not available.
