# Supplementary material for: Novel Co5 and Ni4 Metal Complexes and Ferromagnets by the Combination of 2-Pyridyl Oximes with Polycarboxylic Ligands
Source: Molecules. 2022 Jul 22;27(15):4701. doi: 10.3390/molecules27154701 (PMC9332737; doi:10.3390/molecules27154701)
Supplement: Supplementary file 1 [file molecules-27-04701-s001.zip › molecules-1823195-supplementary.pdf]

Article

Novel Co_5 and Ni_4 Metal Complexes and Ferromagnets by the Combination of 2-Pyridyl Oximes with Polycarboxylic Ligands

Foteini Dimakopoulou [1], Costantinos G. Efthymiou [1], Ciaran O'Malley [1], Andreas Kourtellaris [2], Eleni Moushi [3], Anastasios Tasiopooulos [2], Spyros P. Perlepes [4], Patrick McArdle [1], Ernesto Costa-Villén [5], Julia Mayans [5] and Constantina Papatriantafyllopoulou [1,*]

[1] School of Biological and Chemical Sciences, College of Science and Engineering, National University of Ireland Galway, University Road, H91 TK33 Galway, Ireland; f.dimakopoulou1@nuigalway.ie (F.D.); dinosef@yahoo.com (C.G.E.); c.omalley16@nuigalway.ie (C.O.); patrick.mcardle@nuigalway.ie (P.M.)
[2] Department of Chemistry, University of Cyprus, Nicosia 1678, Cyprus; kourtellaris.andreas@ucy.ac.cy (A.K.); atasio@ucy.ac.cy (A.T.)
[3] Department of Life Sciences, European University of Cyprus, Nicosia 2404, Cyprus; e.moushi@euc.ac.cy
[4] Department of Chemistry, University of Patras, 26504 Patras, Greece; perlepes@upatras.gr
[5] Departament de Química Inorgànica i Orgànica, Secció Inorgànica and Institute of Nanoscience (IN2UB) and Nanotecnology, Universitat de Barcelona, Marti i Franques 1-11, 08028 Barcelona, Spain; ernesto.costa@qi.ub.edu (E.C.-V.); julia.mayans@qi.ub.edu (J.M.)
* Correspondence: constantina.papatriantafyllopo@nuigalway.ie; Tel.: +353-91-493462

Table S1. Selected interatomic distances (Å) and angles for **1**.

**Bonds**			
Ni1-O3	2.018(3)	Ni1-N3	2.019(3)
Ni1-O4	1.988(3)	Ni2-O3	1.814(3)
Ni1-O19	2.272(4)	Ni2-N4	1.846(3)
Ni1-N1	2.098(4)	Ni2-N5	1.886(4)
Ni1-N2	2.057(3)	Ni2-N6	1.855(4)
**Angles**			
N1-Ni1-O19	174.50(15)	N5-Ni2-O3	173.15(14)
N3-Ni1-O4	169.49(13)	N4-Ni2-N6	174.77(15)
N2-Ni1-O3	175.00(14)		

Table S2. Selected interatomic distances (Å) and angles for **2**.

**Bonds**			
Co1-O3	1.951(5)	Co3-O7	1.913(5)
Co1-O9	1.904(5)	Co3-O11	1.925(5)
Co1-N1	1.922(6)	Co4-O7	1.913(5)
Co1-N2	1.885(6)	Co4-O10	1.902(5)
Co1-N3	1.916(6)	Co4-N9	1.914(6)
Co1-N4	1.908(6)	Co4-N10	1.894(6)
Co2-O1	1.907(5)	Co4-N11	1.931(5)

Co2-O9	1.901(5)	Co4-N12	1.899(6)
Co2-N5	1.911(6)	Co5-O6	1.948(5)
Co2-N6	1.901(6)	Co5-O10	1.911(5)
Co2-N7	1.917(6)	Co5-N13	1.921(6)
Co2-N8	1.907(6)	Co5-N14	1.886(6)
Co3-O1	2.104(5)	Co5-N15	1.908(6)
Co3-O4	1.946(5)	Co5-N16	1.908(6)
Co3-O5	1.942(5)		
**Angles**			
O3-Co1-N4	173.9(2)	O4-Co3-O5	108.1(2)
N1-Co1-O9	172.0(2)	O7-Co4-N9	174.5(2)
N2-Co1-N3	178.3(2)	O10-Co4-N11	170.0(2)
O1-Co2-N7	174.0(2)	N10-Co4-N12	176.2(2)
N6-Co2-N8	175.3(3)	O6-Co5-N16	174.1(2)
O9-Co2-N5	170.1(2)	O10-Co5-N13	171.1(2)
O1-Co3-O7	173.50(18)	N14-Co5-N15	178.0(3)

Table S3. Selected interatomic distances (Å) and angles for **3**.

**Bonds**			
Co1-O4	1.947(7)	Co3-N7	2.114(6)
Co1-O6	1.908(6)	Co3-N12	2.114(6)
Co1-O24	1.927(6)	Co4-O12	1.878(8)
Co1-N2	1.900(6)	Co4-O16	1.908(6)
Co1-N4	1.907(7)	Co4-O18	1.913(9)
Co1-N11	1.888(7)	Co4-N6	1.907(7)
Co2-O2	2.004(6)	Co4-N8	1.889(7)
Co2-O20	1.980(6)	Co4-N10	1.840(8)
Co2-O25	2.030(6)	Co5-O8	2.011(6)
Co2-N1	2.058(6)	Co5-O10	1.992(6)
Co2-N9	2.114(7)	Co5-O25	1.986(5)
Co3-O14	1.994(6)	Co5-N3	2.086(6)
Co3-O22	2.004(6)	Co5-N5	2.122(6)
Co3-O25	1.997(5)		
**Angles**			
O4-Co1-N4	171.8(3)	O14-Co3-O25	130.9(3)
O6-Co1-N11	171.7(3)	O12-Co4-N10	169.8(3)
O24-Co1-N2	170.9(3)	O18-Co4-N8	171.2(3)
N1-Co2-N9	176.2(3)	O16-Co4-N6	171.3(3)
O2-Co2-O25	129.1(2)	N3-Co5-N5	175.8(3)
O20-Co2-O25	126.0(3)	O8-Co5-O25	129.9(2)
N7-Co3-N12	176.6(3)	O10-Co5-O25	128.2(2)

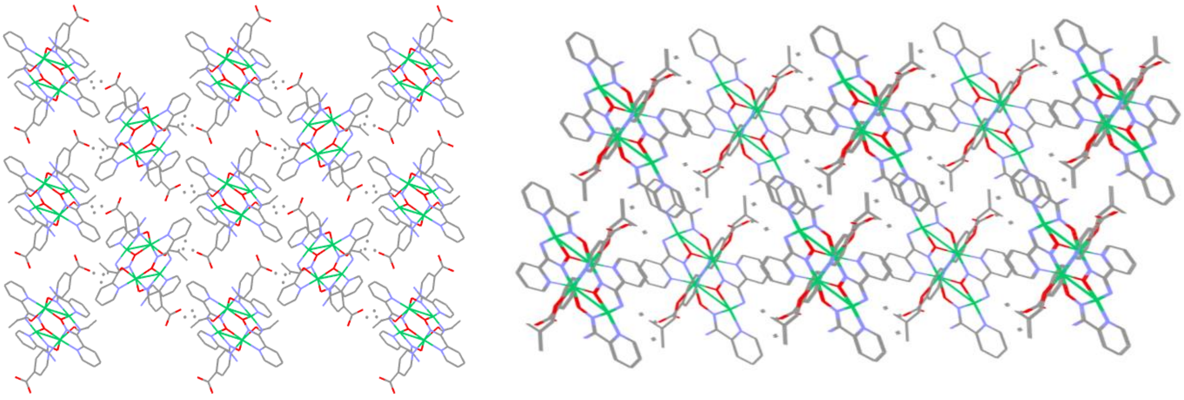

Figure S1. The 2D supramolecular network in **1** coming from the arrangement of Ni4 clusters through H-bonding interactions (left), and the arrangement of two parallel 2D supramolecular planes (right).

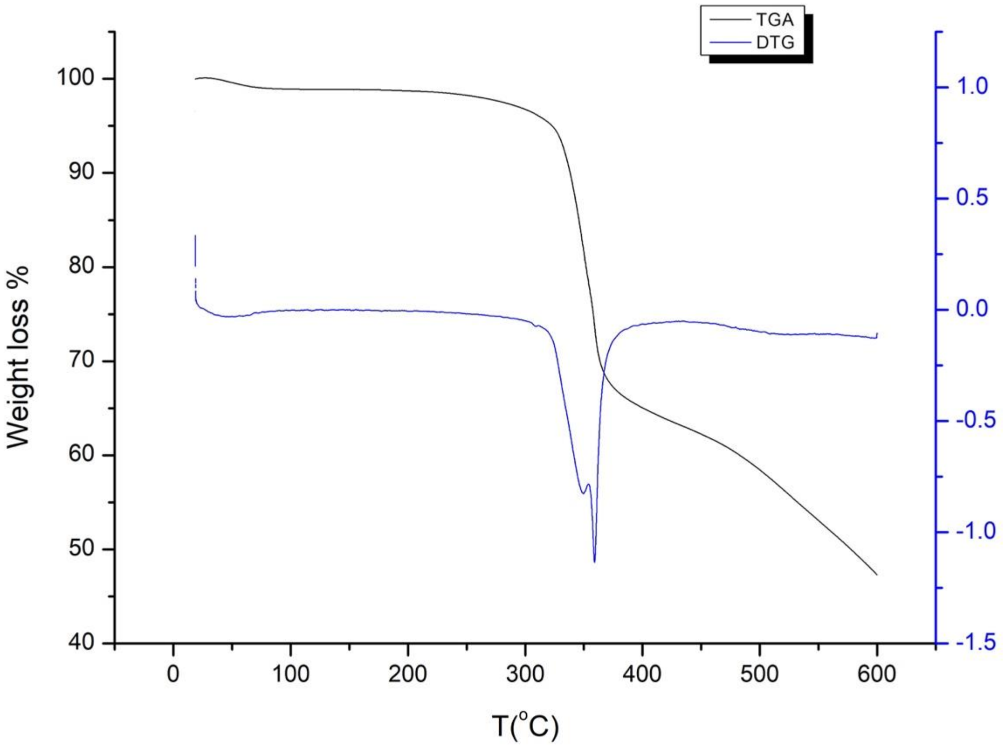

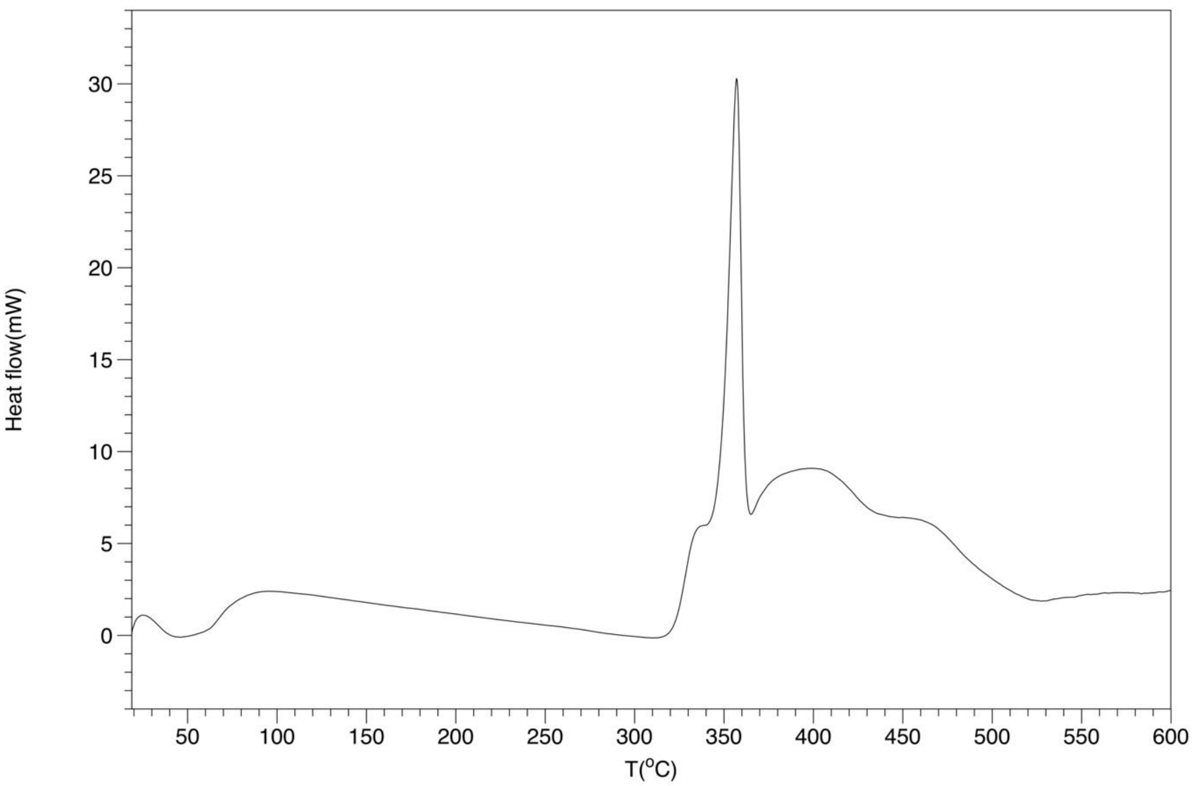

Figure S2. TGA-DTG (top) and DSC curves (bottom) of compound **1**.

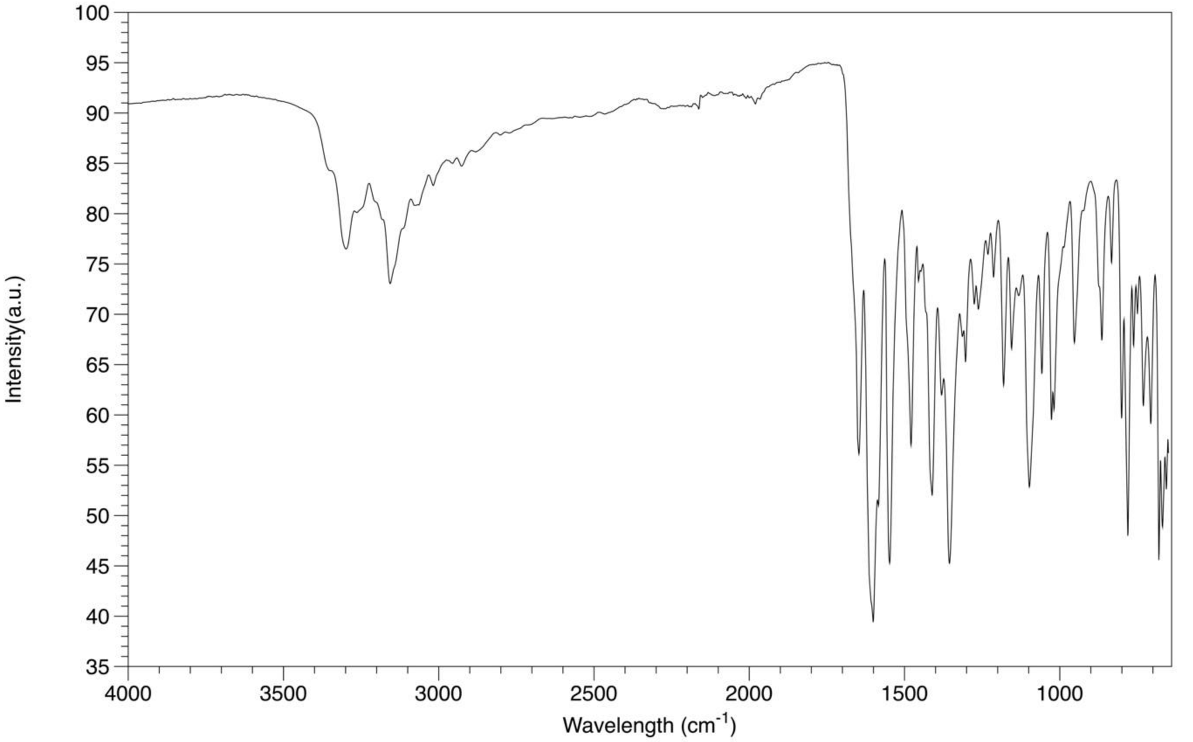

Figure S3. IR spectrum of compound **1**.

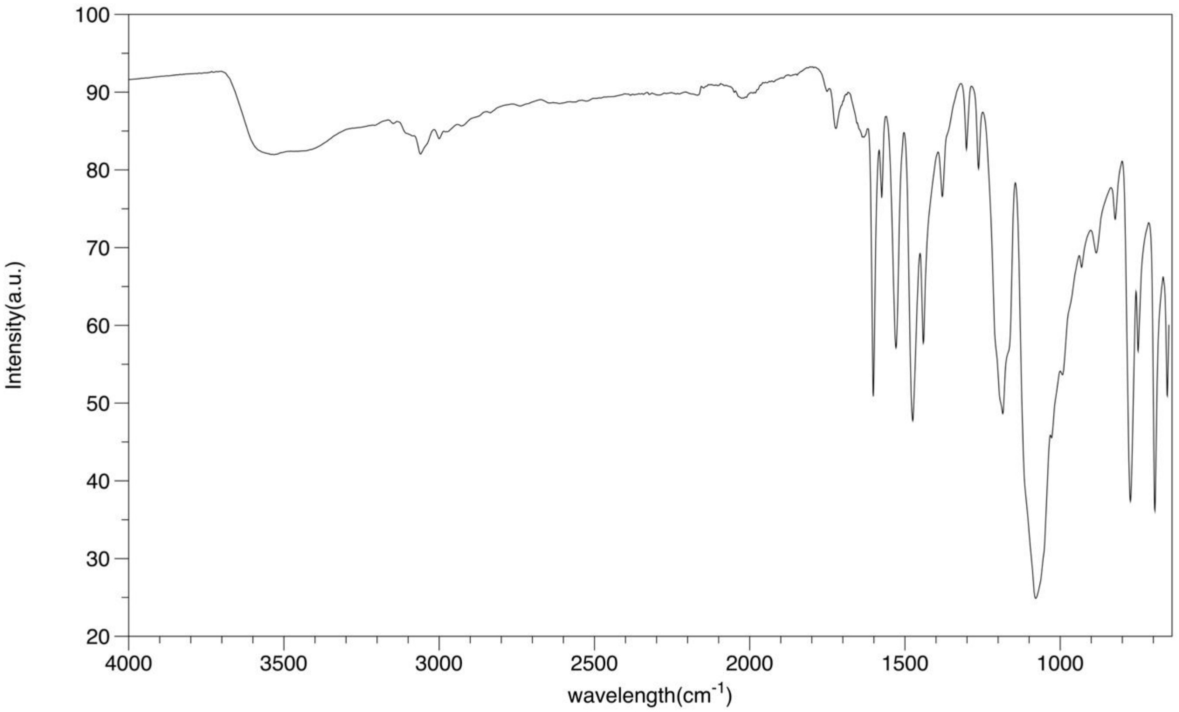

Figure S4. IR spectrum of compound **2**.

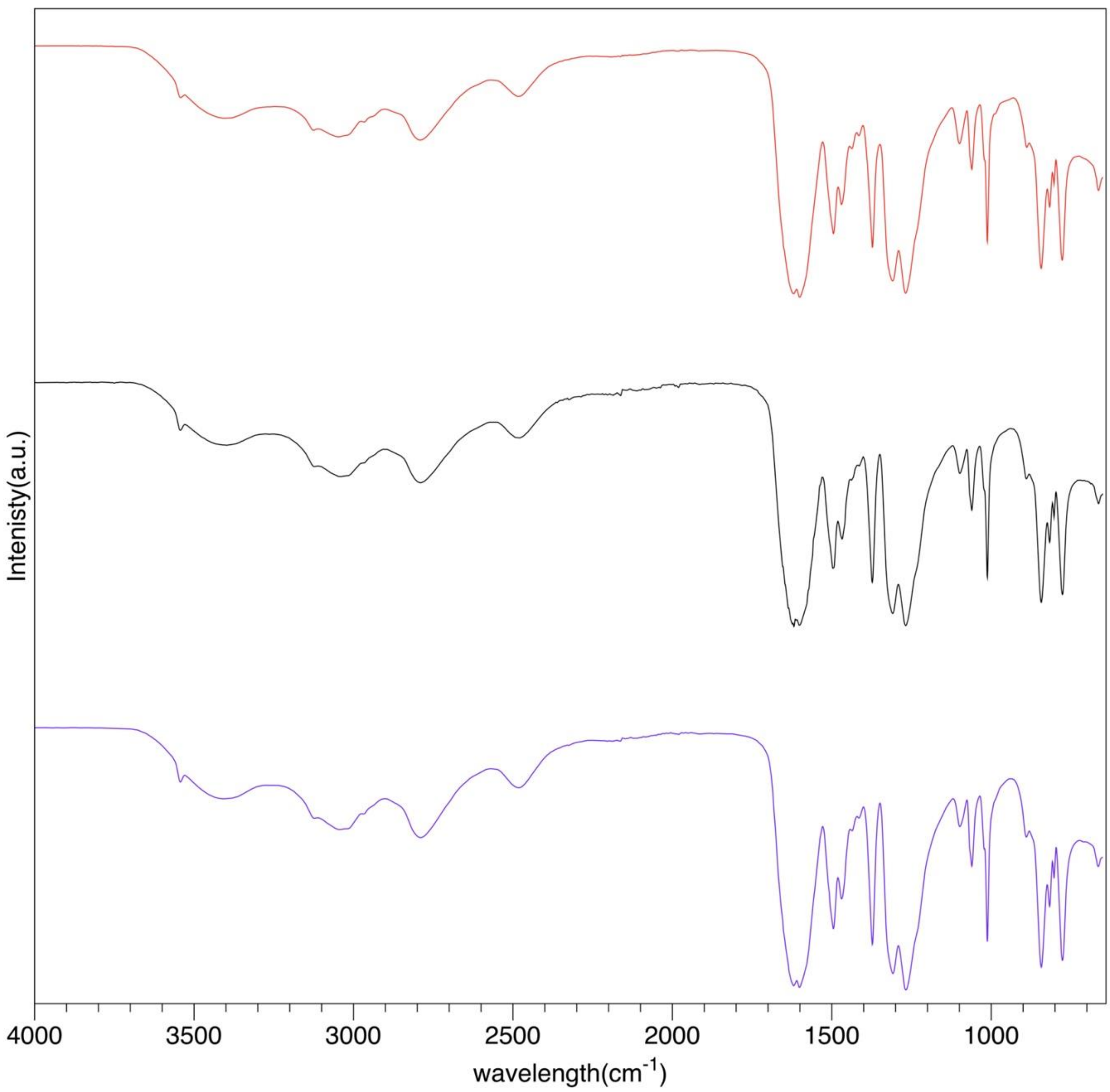

Figure S5. IR spectra of compound **3:** crystals from method A, top; crystals from method B, middle; crystals from Method C, bottom.

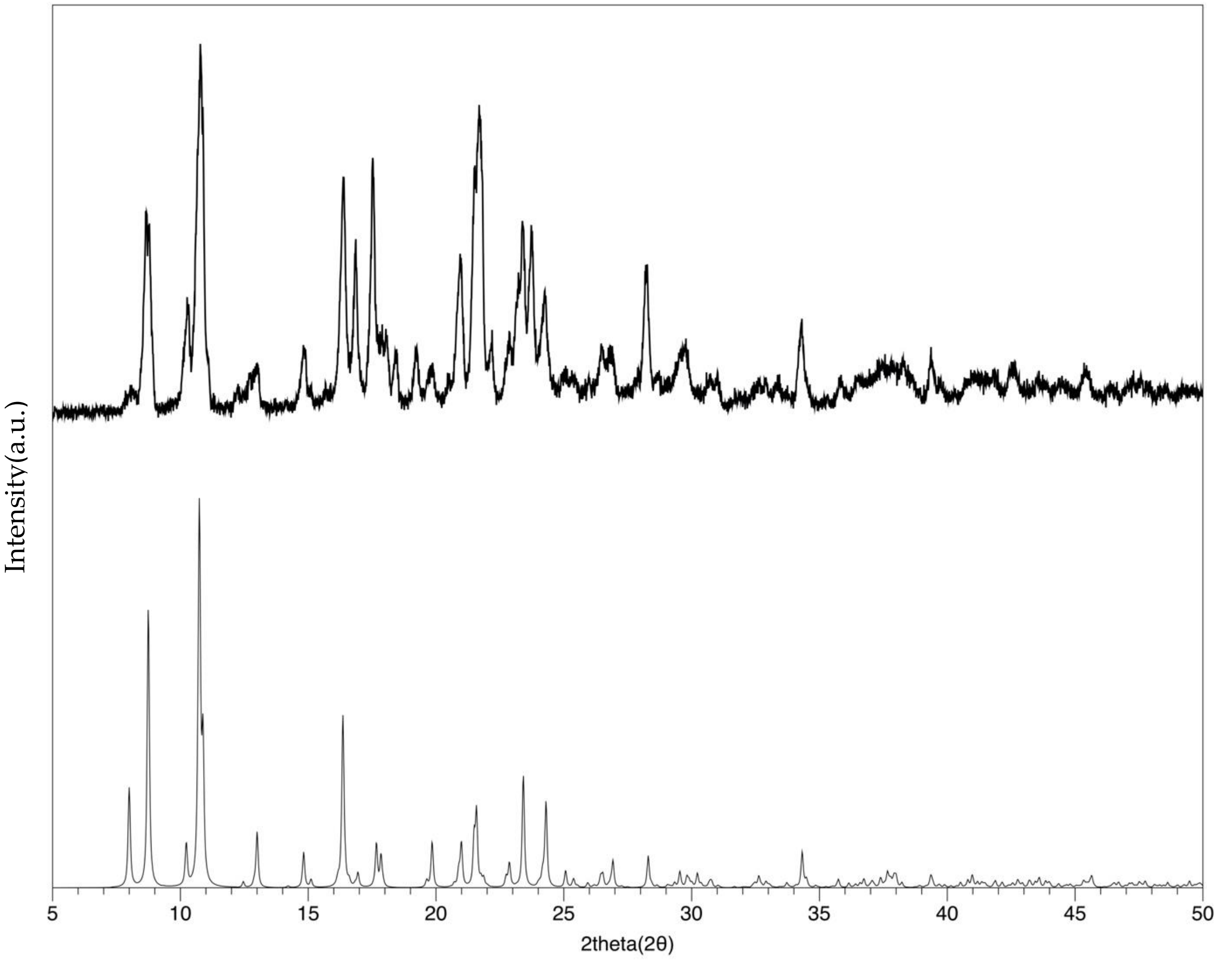

Figure S6. Powder XRD patterns of compound **1**: experimental powder pattern, top; simulated, bottom.
